# Changes in the Structure and Corrosion Protection Ability of Porous Anodic Oxide Films on Pure Al and Al Alloys by Pore Sealing Treatment

**DOI:** 10.3390/ma15238544

**Published:** 2022-11-30

**Authors:** Haruno Yanagimoto, Koki Saito, Hideaki Takahashi, Makoto Chiba

**Affiliations:** National Institute of Technology, Asahikawa College, Asahikawa 071-8142, Japan

**Keywords:** corrosion protection, aluminum alloys, anodic oxide film, electrochemical impedance spectroscopy

## Abstract

It is well known that corrosion protection of pure Al is enormously improved by the formation of porous anodic oxide films and by pore sealing treatment. However, the effects of anodizing and pore sealing on corrosion protection for Al alloys are unclear, because the alloying elements included in Al alloys affect the structure of anodic oxide films. In the present study, porous anodic oxide films are formed on pure Al, 1050-, 3003- and 5052-Al alloys, and pore sealing was carried out in boiling water. Changes in the structure and corrosion protection ability of porous anodic oxide films on pure Al and the Al alloys by pore sealing, were examined by scanning electron microscopy (SEM) and electrochemical impedance spectroscopy (EIS). SEM observation showed that anodic oxide films formed on pure Al have a smooth surface after pore sealing, and that cracks are formed in anodic oxide films on 1050-, 3003- and 5052-aluminum alloys, after pore sealing. Corrosion protection after pore sealing increased with anodizing time on pure Al, but only slightly increased with anodizing time on the Al alloys.

## 1. Introduction

Aluminum is one of the most popular and important metallic materials because of its excellent physical and chemical properties. Six-thousand series of Al alloys (Al-Mg-Si-Cu) are used for automobiles, due to their excellent strength, lightness, corrosion protection, formability and recyclability [1]. Airplane bodies are made of Al-FRP laminates because they have low specific gravity and a high strength-to-weight ratio [2]. Al alloy-cladded pipes are used as outdoor heat exchangers of air-conditioners because of their high heat-conductivities, high processabilities, and stable supply chains [3,4]. 

Corrosion protection of Al and Al-based alloys is relatively poor, and Al alloy industrial products suffer from corrosion under application. Corrosion of Al and Al alloys has been investigated by many researchers [5,6,7,8,9,10,11], and pitting corrosion in Cl^-^ solution [6], stress corrosion cracking [7], fatigue of aircraft body [8], corrosion in alcohol [9], reaction with hot water [10], high temperature oxidation [11], and so on, are the main topics in their research works.

In order to improve the corrosion protection of pure Al and Al alloys, surface treatments, including anodizing, chemical coating, metal plating, painting, organic coating, and spray coating, are applied. In particular, anodizing, which forms porous anodic oxide films in acid solutions, including sulfuric acid, oxalic acid, phosphoric acid and chromic acid, is a popular, simple and effective technique for corrosion protection of pure Al and Al alloys. The porous oxide film formed on pure aluminum has numerous nano-pores perpendicular to the substrate, and a thin barrier layer at the bottom of the pores [12]. The morphology of the anodic porous oxide films is directly correlated with anode potential, and with increasing the anode potential, the number of pores decreases, and the diameter of pores increases [13].

The structure and formation of porous anodic oxide films on Al alloys are different from those on pure Al, because alloying elements are included as solid solution, intermetallic compounds, and inclusions in Al alloys, and they dissolve in the solution preferentially and are incorporated in the oxide film [14,15,16,17]. Cote et al. cast Al-3Mn, Al-5Fe, Al-6.4Mg-3.7Si, and Al-2Cr to allow the growth of intermetallic compounds in Al alloys, and examined the behavior of the intermetallic compound during anodizing in sulfuric acid solution. They found that MnAl_6_ was inert, FeAl_6_ dissolved at the same rate as the matrix, Mg_2_Si was oxidized more rapidly than the matrix with dissolution, CrAl_7_ dissolved more rapidly than the matrix, and that Mn, Si, and Cr in the solid solution were incorporated in the oxide film, while Mg dissolved [14]. They also examined Al-10Cu, Al-10Mg, Al-13Si, Al-Ti, Al-4Fe-8Si, Al-9Mg-3Zn, and Al-9Zn-3Mg casting alloys after anodizing, under the same condition as described above, and found that Si, TiAl_3_, β-AlFeSi particles are incorporated in anodic oxide films, while CuAl_2_ and β-AlMg phases are rapidly oxidized and easily dissolve [15]. Cu, Fe, and Mg in the solid solution were found to largely dissolve. 

Corrosion protection ability of the oxide film on pure Al is enormously improved by pore sealing after anodizing, and this pore sealing has been investigated for many years [18,19,20]. First, S. Setoh and A. Miyata found that corrosion protection of aluminum covered with porous anodic oxide films, becomes much higher on contact with pressurized water vapor [18,19]. Since then, many techniques on pore sealing have been studied, immersion in boiling pure water, Ni-, Cr(VI)-, Li-, Ce-based solutions [20], and so on. 

Pore sealing is generally carried out by immersion in boiling water [21], and recently the mechanism of the hot water sealing was reviewed in detail by S. Ono et al. [22]. At the initial stage of pore sealing, anodic oxide films dissolve in nano-pores to precipitated flaky aluminum hydroxides on the inner wall of pores. Then, precipitation of the hydroxide proceeds to fill the nano-pore, and to form a flake layer on the surface of the oxide film. At the same time, penetration of water through the outermost layer enables oxides to transform to hydroxide. Finally, anodic oxide films, after pore sealing, consist of three layers: (1) an outermost flaky layer, (2) an intermediate hydrated oxide layer, and (3) an innermost oxide layer with hydroxide in nano-pores. The thickness of the outermost and intermediate layers increases with sealing time, while the thickness of the innermost layer decreases. The change in the thickness of the three layers becomes slower with time to reach zero, i.e., steady state.

The chemical reaction during pore sealing in hot water is expressed in Equation (1) [23,24,25,26].

Al_2_O_3_ + (1 + 2x)H_2_O→2AlOOH·xH_2_O (1)

M. Kohda et al. [21] determined the value of x by gravimetry and obtained x = 1–2. S. Ono et al. [22] expected the x-value to be x = 0.3–1.3 and obtained a boehmite-like electron diffraction ring from the outermost layer, formed by pore sealing. H. Takahashi et al. [10] analyzed the structure of films formed by immersing pure aluminum in boiling water by FTIR and obtained the spectrum of pseudo-boehmite. 

As described above, alloying elements in Al alloys either dissolve preferentially in the solution, or are incorporated in the oxide film during anodizing, so that the structure of the oxide film formed on Al alloys is different from that on pure Al. Thus, the mechanism of the pore sealing of anodic oxide films on Al alloys, may be affected significantly with chemical composition and phases of alloying elements in the Al alloy. Effects of pore sealing of anodic oxide films on the corrosion protection are studied much less on Al alloys than on pure Al. Y. Hara et al. [27] examined the corrosion protection of 1xxx-Al alloys, including Bi and Sn in alcohol at 415 K, and found that corrosion of anodized/pore-sealed specimens proceeds more rapidly than that of electropolished specimens. This is due to the crack formation in the anodic oxide film, by the compressive stress with Bi and Sn incorporated in the anodic oxide film. M. Kayashima et al. [28] studied the crack formation in porous anodic oxide films by heating and found that the cracking temperature decreases with increasing film thickness and pore filling time in hot water.

In the present investigation, pure Al, 1050-, 3003- and 5052-Al alloys were anodized in oxalic acid solutions to form porous anodic oxide films, and then pores were sealed in boiling water. Surface and corrosion behavior of the specimens after anodizing and pore sealing were examined by SEM and EIS.

## 2. Experimental

Pure Al, AA1050-, 3003- and 5052-Al alloys were used as specimens in this study. The chemical composition and the thickness of specimens are summarized in Table 1. These specimens were cut into 17.5 mm × 30 mm and then electropolished in 78 vol.%-CH_3_COOH/22 vol.%-HClO_4_ solution with a constant potential of 30 V at 273 K for 15 s, as a pretreatment. Pretreated specimens were anodized in 2 wt% -(COOH)_2_ solution with a constant c. d. of 200 Am^−2^ at 313 K for *t_a_* = 1800 and 3600 s. After anodizing, the specimen was rinsed with pure water, and then put in a test tube with 100 mL of pure water. Pore sealing was carried out by immersing the test tube in an oil bath at 373 K for *t_s_* = 1200 s. Surfaces of specimens after electropolishing, anodizing and pore sealing were observed by SEM (JEOL, JSM-6510LA, Tokyo, Japan). Vertical cross sections of all the specimens after anodizing for *t_a_* = 1800 and 3600 s, and pore sealing for *t_p_* = 1200 s, were also observed by SEM. Prior to SEM observation, all the specimens were coated with a thin layer of Au. To clarify the mechanism on the change in the film structure during pore sealing, pure Al and Al alloy specimens with *t_a_* = 3600 s were heated at *T_h_* = 373 K for *t_h_* = 120 s in air atmosphere, and the specimen surface was observed by SEM. 

**Figure 1:**
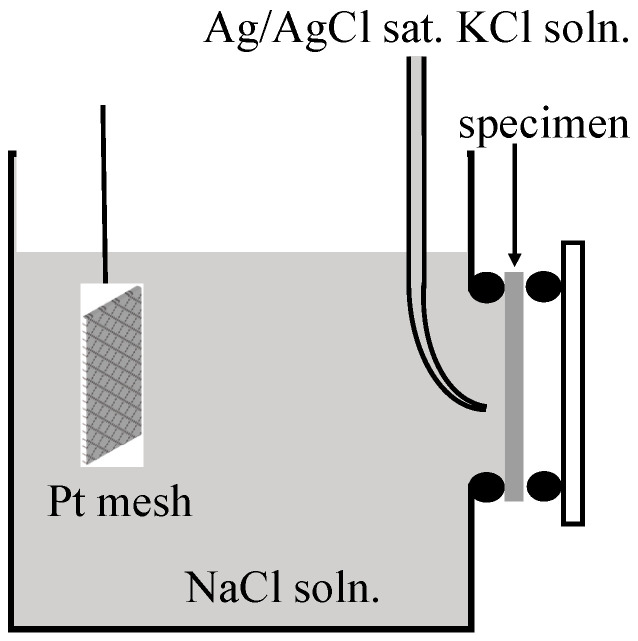
Schematic diagram of electrochemical cell used for electrochemical impedance spectroscopy.

Corrosion protection of specimens after pore sealing was evaluated by EIS (Hokuto-Denko, Hz-7000, Tokyo, Japan) in 2.0 kmol m^−3^-NaCl solution, after bubbling N_2_ gas for 1200 s using the electrochemical cell shown in Figure 1. Pt mesh and Ag/AgCl in saturated KCl solution were employed as counter and reference electrodes, respectively. Measurements were carried out by applying a sinusoidal wave of 10 mV between 10 Hz–20 kHz.

## 3. Results

### 3.1. Growth of Anodic Oxide Films on Pure Al and Al Alloys during Anodizing

**Figure 2:**
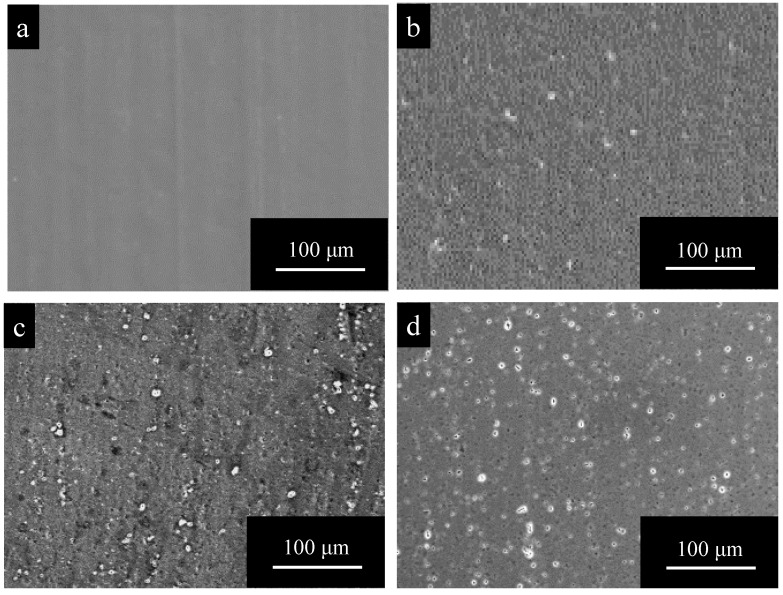
SEM images of surfaces of (**a**) pure Al, (**b**) 1050-al alloy, (**c**) 3003-Al alloy, and (**d**) 5052-Al alloy specimens after electropolishing (*t_a_* = 0). (**c**) enlarged to 500% can be found in Figure A1 in the Appendix A.

Figure 2 shows SEM images of surfaces of (a) pure Al, (b) 1050-al alloy, (c) 3003-Al alloy, and (d) 5052-Al alloy specimens after electropolishing (*t_a_* = 0). The surface of pure Al is very smooth and there appear to be no imperfections (Figure 2a). The surface of Al alloys is relatively rough, and there are many white particles and pits with a diameter of several μm (Figure 2b–d). The number of particles and pits on 3003- and 5052- Al alloys are larger than that on 1050-Al alloy.

**Figure 3:**
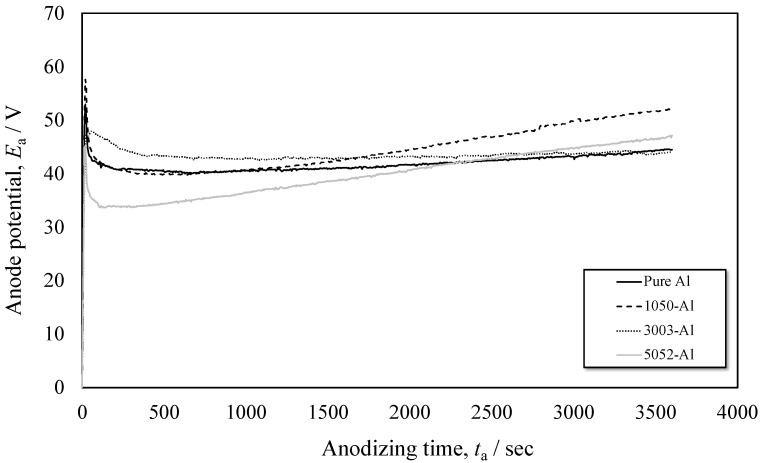
Anode potential transients (*E_a_* vs. *t_a_*) during anodizing of pure Al, 1050-, 3003- and 5052-Al alloys with a constant c.d. of 200 Am^−2^ in 2 wt% -(COOH)_2_ solution at 313 K.

Figure 3 shows anode potential transients (*E_a_* vs. *t_a_*) during anodizing of pure Al, 1050-, 3003- and 5052-Al alloys with a constant c.d. of 200 Am^−2^ in 2 wt% -(COOH)_2_ solution at 313 K. On pure Al, anode potential increases to ca. 50 V at the very initial stage, and decreases to reach a steady value of ca. 40 V at *t_a_* = 250 s. This is a typical potential transient during growth of porous anodic oxide films [13], [29]. The anode potential transient on 1050-Al alloy is like that on pure Al before *t_a_* = 250 s, but beyond *t_a_* = 250 s potential increases gradually with *t_a_* to reach *E_a_* = 50 V at *t_a_* = 3600 s. On 3003-Al alloy, the potential decreases slowly after an initial potential peak of *E_a_* = 45 V, to reach a steady value of *E_a_* = 40 V at *t_a_* = 500 s. On 5052-Al alloy, after a sharp initial potential peak, the potential gradually increases to *E_a_* = 43 V at *t_a_* = 3600 s, through a minimum value of *E_a_* = 33 V at *t_a_* = 100 s.

**Figure 4:**
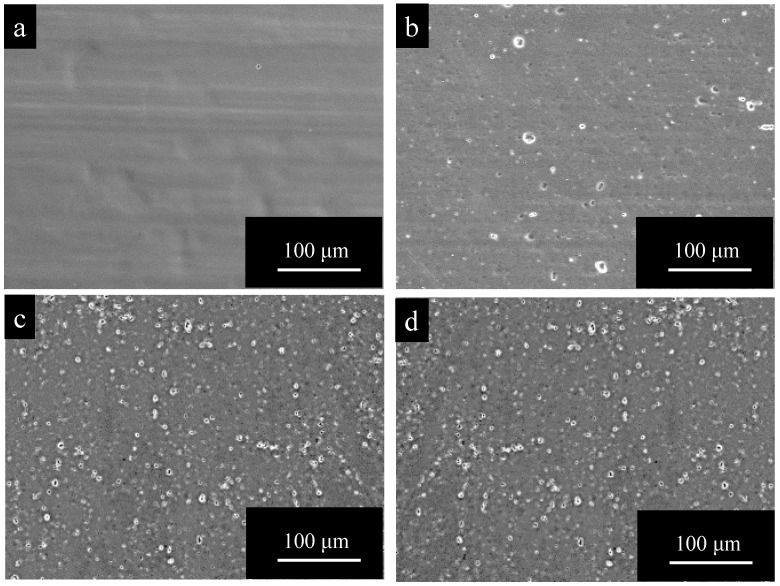
SEM images of surfaces of (**a**) pure Al, (**b**) 1050-al alloy, (**c**) 3003-Al alloy, and (**d**) 5052-Al alloy specimens after anodizing for *t_a_* = 1800 s. Anodizing conditions are described in Figure 3.

Figure 4 shows SEM images of surfaces of (a) pure Al, (b) 1050-al alloy, (c) 3003-Al alloy, and (d) 5052-Al alloy specimens, after anodizing for *t_a_* = 1800 s. The surface of anodic oxide films after anodizing for *t_a_* = 1800 s is smooth on pure Al and has many pits on the Al alloys. Comparing Figure 4 with Figure 2, one can see that surface morphology is quite consistent on each specimen with *t_a_* = 1800 s and with *t_a_* = 0.

**Figure 5:**
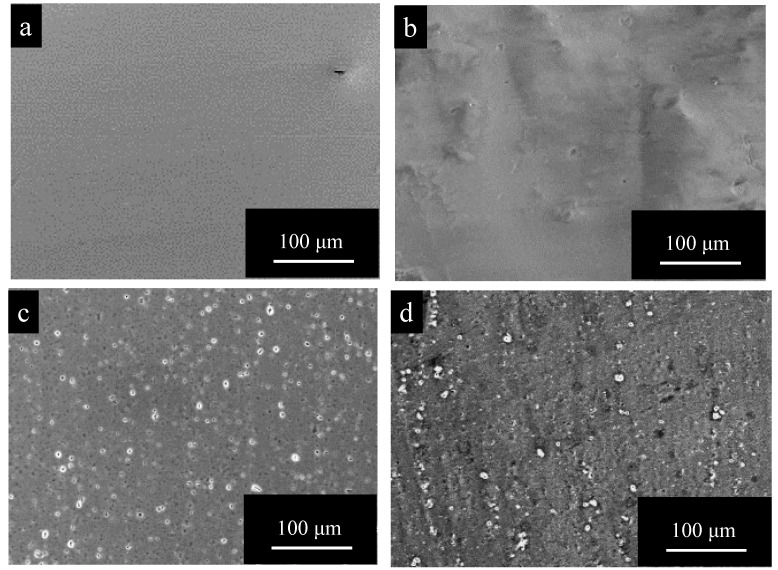
SEM images of surfaces of (**a**) pure Al, (**b**) 1050-al alloy, (**c**) 3003-Al alloy, and (**d**) 5052-Al alloy specimens after anodizing for *t_a_* = 3600 s. Anodizing conditions are described in Figure 3.

Figure 5 shows SEM images of surfaces of (a) pure Al, (b) 1050-al alloy, (c) 3003-Al alloy, and (d) 5052-Al alloy specimens, after anodizing for *t_a_* = 3600 s. Surface morphology of each specimen with *t_a_* = 3600 s is like the specimen of *t_a_* = 0 and 1800 s. 

**Figure 6:**
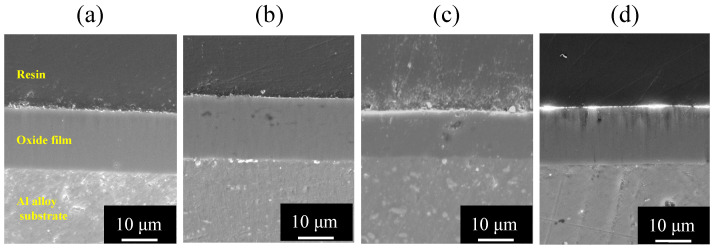
SEM images of the vertical cross section of (**a**) pure Al, (**b**) 1050-al alloy, (**c**) 3003-Al alloy, and (**d**) 5052-Al alloy specimens after anodizing for *t_a_* = 1800 s. Anodizing conditions are described in Figure 3.

Figure 6 shows SEM images of the vertical cross section of (a) pure Al, (b) 1050-al alloy, (c) 3003-Al alloy, and (d) 5052-Al alloy specimens after anodizing for *t_a_* = 1800 s.

Each image consists of three layers, as follows: a top layer of embedding resin, a middle layer of oxide film, and a bottom layer of metal substrate. The thickness of oxide films on pure Al, 1050-Al alloy, and 5052-Al alloy is about δ = 15 μm and is larger than that on 3003-Al alloy. 

**Figure 7:**
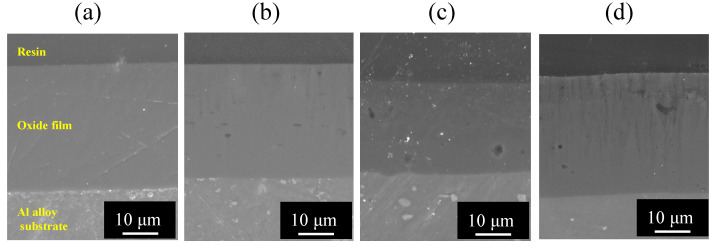
SEM images of the vertical cross section of (**a**) pure Al, (**b**) 1050-Al alloy, (**c**) 3003-Al alloy, and (**d**) 5052-Al alloy specimens with *t_a_* = 3600 s. Anodizing conditions are described in Figure 3.

Figure 7 shows SEM images of the vertical cross section of (a) pure Al, (b) 1050-Al alloy, (c) 3003-Al alloy, and (d) 5052-Al alloy specimens with *t_a_* = 3600 s. The thickness of oxide films on pure Al, 1050-Al alloy, and 5052-Al alloy is about δ = 30 μm, and is larger than that on 3003-Al alloy. In the oxide film formed on 3003-Al alloy, a relatively large number of white particles are incorporated. It can be seen from Figure 6 and Figure 7 that there are light grey patterns in the substrate of 1050-, 3003-, and 5052-Al alloys, suggesting second phases in the alloys. There are also cavities in the oxide film on 3003-, and 5052-Al alloys, and this may be due to the results of preferential dissolution of the second phase in the alloys. 

**Figure 8:**
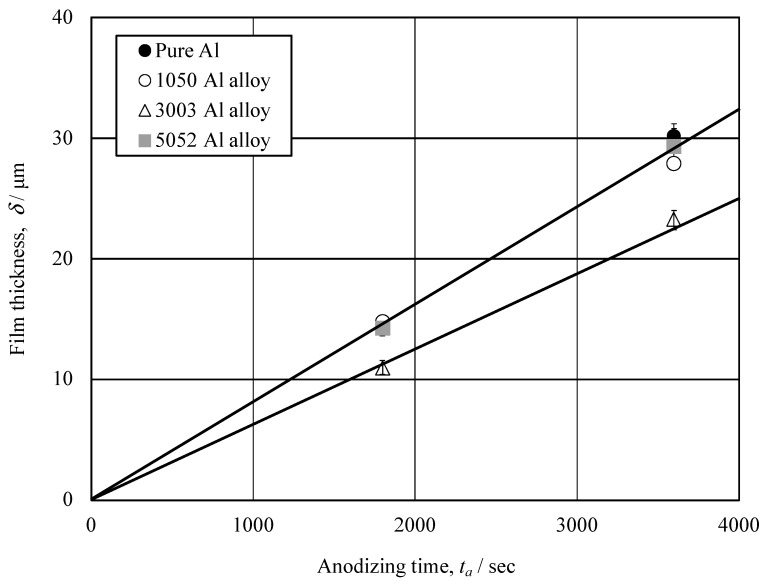
Relationship between film thickness, *δ*, and anodizing time, *t_a_*, obtained for pure Al and Al alloys. Anodizing conditions are described in Figure 3.

Figure 8 shows the relationship between film thickness, δ, and anodizing time, *t_a_*, obtained for pure Al and Al alloys. It can be seen from Figure 8, that δ is proportional to *t_a_* on each specimen, and the proportional constant is 8 nm/s on pure Al, 1050- and 5052-Al alloys and 6 nm/s on 3003-Al alloy.

### 3.2. Surface Morphology Change of Pure Al and Al Alloys by Pore Sealing

**Figure 9:**
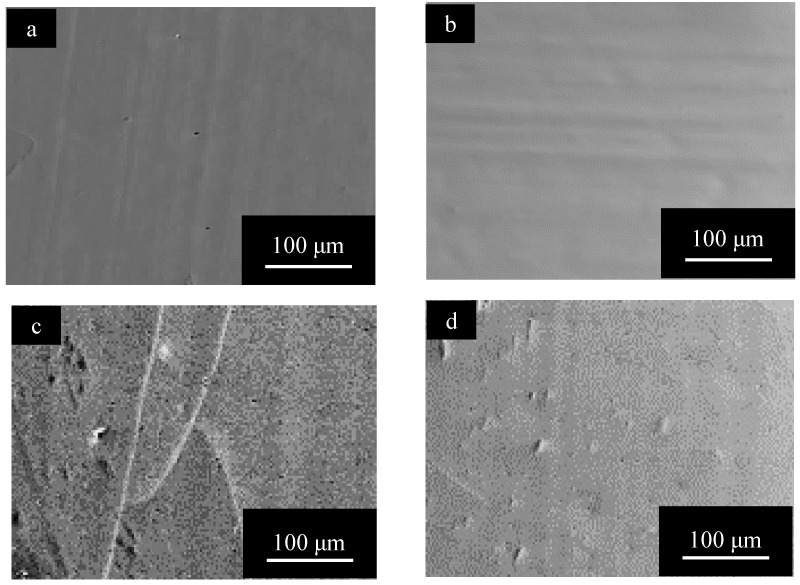
SEM images of the surface of specimens with *t_a_* = 1800 s and *t_s_* = 1200 s, obtained for (**a**) pure Al, (**b**) 1050-Al alloy, (**c**) 3003-Al alloy, and (**d**) 5052-Al alloy. Anodizing conditions are described in Figure 3. Pore sealing was carried out by immersion in boiling water for *t_p_* = 1200 s.

Figure 9 shows SEM images of the surface of specimens with *t_a_* = 1800 s and *t_s_* = 1200 s, obtained for (a) pure Al, (b) 1050-Al alloy, (c) 3003-Al alloy, and (d) 5052-Al alloy. Comparing Figure 9 with Figure 4, the surface morphology after anodizing for *t_a_* = 1800 s shows no change by pore sealing on pure Al, being smooth and having no imperfections [6], and on all the Al alloy specimens, the number of pits decreases enormously by pore sealing. A special feature of the surface of the anodic oxide film on 3003-Al alloy, is the formation of a network of cracks (Figure 9c). 

**Figure 10:**
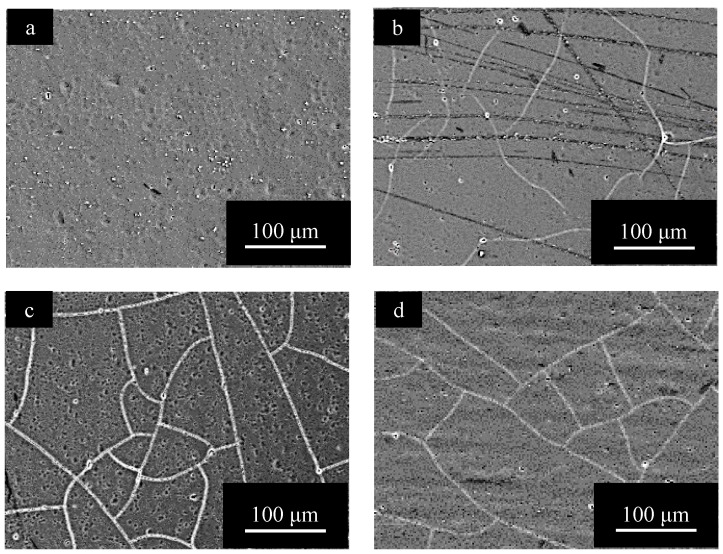
SEM images of the surface of specimens with *t_a_* = 3600 s and *t_s_* = 1200 s, obtained for (**a**) pure Al, (**b**) 1050-Al alloy, (**c**) 3003-Al alloy, and (**d**) 5052-Al alloy. Anodizing condition is described in Figure 3, and pore sealing condition is described in Figure 9.

Figure 10 shows SEM images of the surface of specimens with *t_a_* = 3600 s and *t_s_* = 1200 s, obtained for (a) pure Al, (b) 1050-Al alloy, (c) 3003-Al alloy, and (d) 5052-Al alloy. The surface of pure Al is relatively rough and there appear to be fine needle-like flakes. On all the Al-alloy specimens, there are networks of cracks, and the width of cracks is larger in the order of:

3003-Al alloy > 5052-Al alloy > 1050-Al alloy(2)

**Figure 11:**
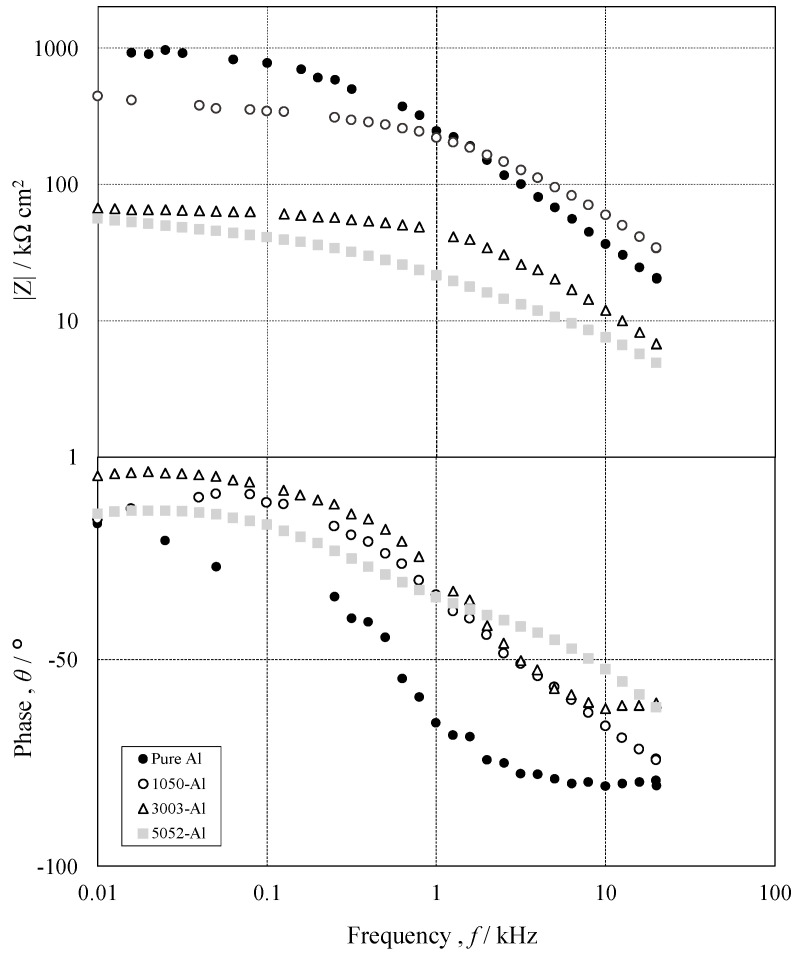
Bode plot on pure Al (●), 1050- (○), 3003- (△) and 5052- (□) Al alloys with *t_a_* = 3600 s and *t_s_* = 1200 s, obtained by EIS measurements. EIS measurements were carried out in 2.0 kmol m^−3^-NaCl solution after bubbling N_2_ gas for 1200 s.

To examine the effect of heating during pore sealing on the formation of cracks, pure Al and Al alloys anodized for *t_a_* = 3600 s were heated in air atmosphere, at *T_h_* = 373 K for *t_h_* = 1200 s. Figure 11 shows a SEM image of the surface of anodized (a) pure Al, (b) 1015-Al alloy, (c) 3003-Al-alloy, and (d) 5052-Al alloy, obtained by heating under the condition described above. The pure Al has a smooth surface without pits (Figure 11a), and this is comparable to Figure 10a. There are many pits on the Al alloy specimens after heating in air (Figure 10b–d), similar to on those after anodizing (Figure 5b–d). The number of pits on 3003-Al alloy is much larger than that on 1015-Al alloy and 5052-Al alloy, and this is consistent with Figure 5.

In conclusion, heating in air atmosphere causes no change in the structure of anodic oxide films, and this is quite unlike the structural change of crack formation by pore filling in boiling water, as shown in Figure 10b–d. This will be discussed in Section 4.2. 

### 3.3. Corrosion Protection of Pure Al and Al Alloys after Anodizing and Pore Sealing

In order to evaluate the effect of pore sealing after anodizing, on the corrosion protection of pure Al and 1050-, 3003- and 5052 Al alloys, EIS was applied in 2.0 kmol m^−3^-NaCl solution. Figure 11 shows Bode plots on pure Al (●), 1050- (○), 3003- (△) and 5052- (□) Al alloys with *t_a_* = 3600 s and *t_s_* = 1200 s, obtained by EIS measurements. The Bode plots on all the specimens consist of two typical frequency regions, as follows: *f* = 0.01–0.5 kHz and *f* = 0.5–20 kHz. In the low frequency region, impedance, *Z*, does not depend on *f*, and phase shift, Δθ, remains around zero. In the high frequency region, log *Z* vs. log *f* shows a straight line with a slope of less than −1, and Δθ decreases with *f* to reach around −90°. The *Z* value in the low frequency region is in the order of

Pure Al > 1050-Al alloy >> 3003-Al alloy = 5052-Al alloy(3)

**Figure 12:**
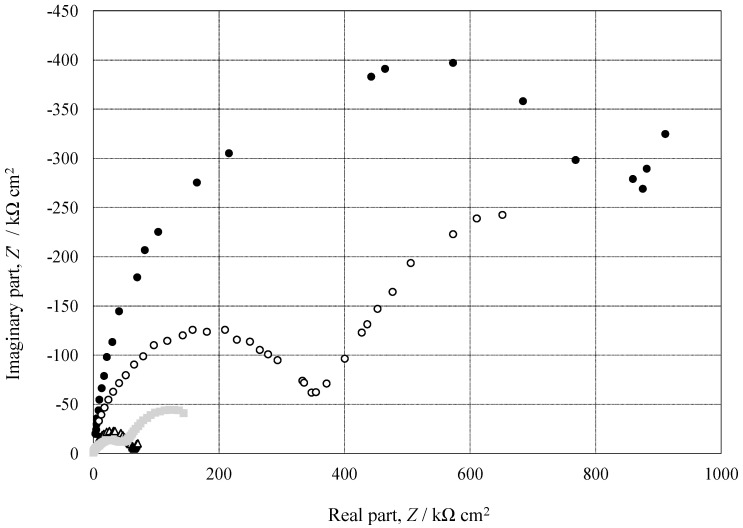
Nyquist plot on pure Al (●), 1050- (○), 3003- (△) and 5052- (□) Al alloys with *t_a_* = 3600 s and *t_s_* = 1200 s, obtained by EIS measurements. EIS measurements were carried out in 2.0 kmol m^−3^-NaCl solution after bubbling N_2_ gas for 1200 s.

Figure 12 shows Nyquist plots on pure Al (●), 1050- (○), 3003- (△) and 5052- (□) Al alloys with *t_a_* = 3600 s and *t_s_* = 1200 s. Nyquist plots on all the specimens consist of two deformed semi-circles with different diameters, and the diameters of both circles are in the order of

Pure Al > 1050-Al alloy > 3003-Al alloy = 5052-Al alloy(4)

## 4. Discussion

### 4.1. Mechanism on the Growth of Anodic Oxide Films during Anodizing of Pure Al and Al Alloys

As shown in Figure 4 and Figure 5, oxide formation during anodizing is quite different between pure Al and Al alloys. Porous oxide films are formed uniformly without any imperfections on pure Al (Figure 4a and Figure 5a). While oxide films, including pits and small particles, are formed on Al alloys (Figure 4b–d and Figure 5b–d). This is discussed below.

As shown in Table 1, 1050-Al alloy includes small amounts of Fe (0.36 mass-%) and Si (0.1 mass-%), as alloying elements. Fe exclusively exists as intermetallic compounds, Al_3_Fe, forming second phases, and Si exists as a solid solution in α-phase and as Si particles. 3003-Al alloy includes relatively large amounts of alloying elements as follows: Mn (1.19 wt%), Fe (0.57 wt%), Si (0.27 wt%), and Cu (0.14 wt%). Mn is included as a solid solution in α-phase and as an intermetallic compound, Al_6_Mn. Cu is included as a solid solution in α-phase and as an intermetallic compound, Al_2_Cu. 5052-Al alloy includes 2.59%-Mg and 0.2%-Cr, as well as small amounts of Si and Fe. Mg is included as a solid solution in α-phase and in β-phase of AlMg. Cr is included as Al_7_Cr [14,15].

As shown in Figure 2, there are many pits on the surface of Al alloys after electropolishing (*t_a_* = 0), while there is no pit on pure Al. This may be the result of localized dissolution of the intermetallic compounds and β-phase, during electropolishing. Possibly, they are detached from the α-phase by the dissolution of α-phase around them. J. Zahavi et al. [30] showed that Al alloys, including 0.4%-and 1.0%-Fe, have surfaces with patches of Al_3_F, and cavities formed by detachment of Al_3_Fe.

During anodizing, the number of patches of Al_3_Fe on the surface, decreases with anodizing time to develop cavities [30], and the second phase of Al/Fe is oxidized more rapidly [31]. This may deduce that the number of pits on the surface of Al alloys with *t_a_* = 1800 and 3600 s, is larger than that with *t_a_* = 0 (see Figure 2, Figure 4 and Figure 5).

Al_6_Mn included in 3003-Al alloy is inert during anodizing, leading to incorporation of itself in the anodic oxide film [14], but Figure 6c and Figure 7c show no evidence of the incorporation. This is probably because Mn in 3003-Al alloy is included as a solid solution in α-phase. Cu as solid solution and as Al_2_Cu in 3003-Al alloy, is rapidly oxidized and dissolved in the solution [15], and this may cause a low growth rate of the film on the alloy (see Figure 3). Additionally, cavities in the oxide film on 3003-Al alloy may be due to the preferential dissolution of Al_2_Cu (see Figure 6c and Figure 7c).

Mg included as β-AlMg phase in 5052-Al alloy, dissolves in the solution during anodizing, leading to the formation of voids [15], and cavities in the oxide films formed on 5052, may be caused by the preferential dissolution of β-AlMg phase (see Figure 7d).

1050-, 3003-, and 5052-Al alloys possibly form three-element constituent intermetallic compounds, β-AlFeSi phase, and this is inert during anodizing, leading to the incorporation in the oxide films [15]. In this study, the evidence of the effect of β-AlFeSi phase was not observed.

Conclusively, anodic oxide films formed on 1050-, 3003-, and 5052-Al alloys have many pits and cavities, and incorporated metallic compounds.

### 4.2. Structural Change of Anodic Oxide Films during Pore Sealing

During immersion, pure Al covered with porous anodic oxide films in boiling water, hydro-oxy-oxides, and AlOOH⋅xH_2_O, is formed to fill nano-pores, sealing them. On 1050-, 3003-, and 5052-Al alloys, cracking occurs in the oxide film during pore sealing. The crack formation during pore sealing can be explained by either (1) a tensile stress generated by the difference in the heat expansion coefficient, between anodic oxide films and the Al alloy substrate, or (2) a compressive stress by the formation of hydro-oxy-oxides in imperfections of anodic oxide films. 

**Figure 13:**
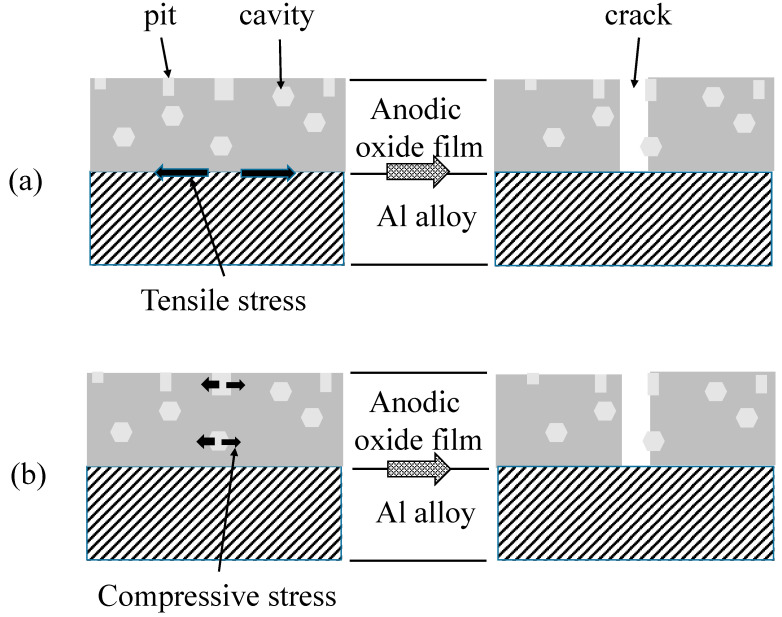
Schematic model of crack formation during pore filling.(**a**) Tensile stress at the interphase between the anodic oxide film and the Al alloy substrate. (**b**) Compressive stress by the formation of hydroxides in pits and cavities.

Heat expansion coefficients of Al and Alumina are 24 × 10^−6^ K^−1^ and 7 × 10^−6^ K^−1^, respectively. One can easily understand that the Al alloy substrate expands to be three times larger than anodic oxide films, during pore sealing in hot water, so that a tensile stress is applied at the interphase between the substrate and the oxide film (Figure 13a, left). Pits and cavities in the oxide film may be the origins of a crack, because of a weakness in physical strength (Figure 13a, right). 

During pore sealing, hydroxides are formed in pits and cavities in anodic oxide films, in addition to nano-pores. Assuming the density of anodic oxide films and hydroxide formed to be 3.0 and 2.4, and the chemical composition of the oxide film and the hydroxide to be Al_2_O_3_and Al_2_O_3_·2H_2_O, the hydration causes a volume expansion of about 8% (=(2.4 × 138)/(3.0 × 102) − 1) [21]. Thus, the formation of hydroxides in pits and cavities may cause a compressive stress from inside to outside (Figure 13b, left), leading to cracking in the oxide film (Figure 13b, right).

**Figure 14:**
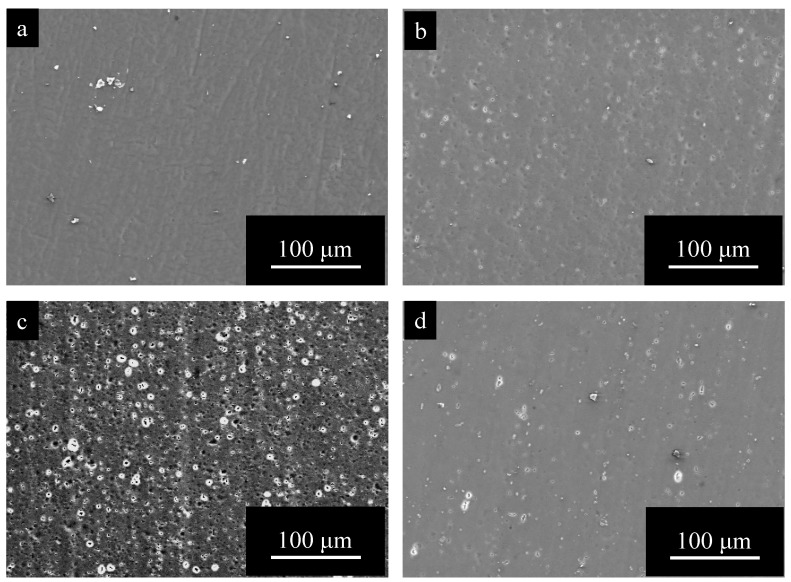
SEM image of the surface of (**a**) pure Al, (**b**) 1050-Al alloy, (**c**) 3003-Al-alloy, and (**d**) 5052-Al alloy, obtained by heating at *T_h_* = 373 K for 1200 s in air after anodizing for *t_a_* = 3600 s.

In order to distinguish the crack formation between the heat expansion mechanism and hydroxide formation mechanism, pure Al and Al alloy specimens with *t_a_* = 3600 s were heated in air atmosphere at *T_h_* = 373 K for *t_h_* = 1200 s. Figure 14 shows SEM images of (a) pure Al, (b) 1050-Al alloy, (c) 3003-Al-alloy, and (d) 5052-Al alloy with *t_a_* = 3600 s and *t_h_* = 1200 s. The pure Al has a smooth surface without pits (Figure 13a), and this is comparable to Figure 5a. There are many pits on the Al alloy specimens after heating in air (Figure 13b–d), such as after anodizing (Figure 5b–d). The authors emphasize here that no cracking occurs from heating at 373 K in air, as opposed to immersion in boiling water. Consequently, crack formation during pore sealing may occur by the formation of hydroxides, rather than by the tensile stress at the interphase between the oxide film and the substrate.

S-M. Moon et al. reported that stresses are generated in anodic oxide films formed on pure Al, and that the stress increases with growth of anodic oxide films by the annihilation of aluminum vacancies, and the generation of oxygen vacancies at the aluminum/oxide interface [32]. Additionally, they found that the direction of stress, generated by the anodizing of Al alloy, changes by pore sealing treatment after anodizing. R. S. Alwitt et al. measured stresses applied between anodic oxide films and the substrate of pure Al, and found that a tensile stress applied to anodic oxide films after anodizing changes to a compressive stress, by pore sealing in boiling water [33]. Compressive stresses may be generated by the formation of hydro-oxy-oxides in nano-pores of anodic oxide films. However, it seems that the stress is not high enough to form cracks in anodic oxide films on pure Al, during pore sealing. W. Liu et al. [25] studied pore sealing of 2024-Al alloy by different techniques: (1) self-sealing (immersion in de-ionized water at ambient temperature for 1 week), (2) boiling water sealing (immersion in boiling water for 1800 s), (3) nickel fluoride sealing (immersion in NiF solution at 298 K and in de-ionized water at 333 K for 900 s), and (4) dichromate sealing (immersion in K_2_Cr_2_O_7_ solution at 363–368 K for 1800 s). They found the formation of cracks after self-sealing, boiling water sealing, and nickel fluoride sealing, but no crack formation after dichromate sealing. S. Wang et al. examined the change in the structure of anodic oxide films formed on 2024-T3 Al alloy, by pore sealing in a phytic acid (C_6_H_6_(H_2_PO_4_)_6_) solution at 363 K, and found that cracks are formed [34]. 

Conclusively, cracking in anodic oxide films during pore sealing depends on the kinds of Al alloys and substances filling pores, rather than on temperature. 

### 4.3. Corrosion Protection Change by Pore Sealing

**Figure 15:**
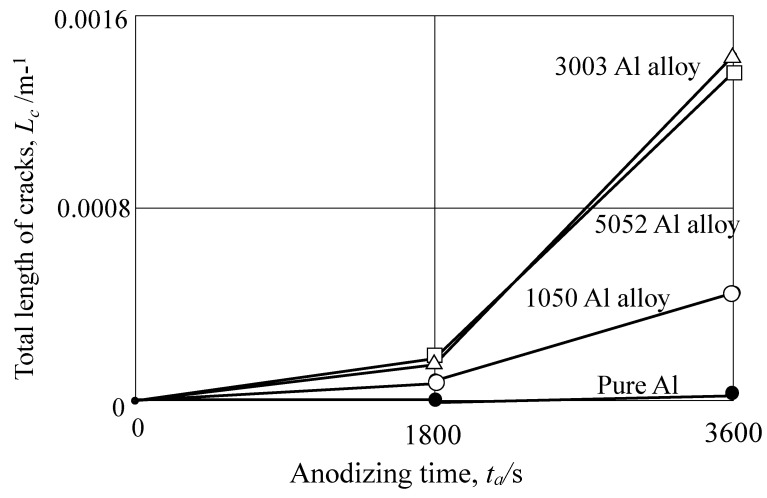
Relationship between the total length of cracks, *L_c_*: m/m^2^ and anodizing time, *t_a_*, obtained for pure Al, 1050-Al alloy, 3003-Al alloy, and 5052-Al alloy. Anodizing condition is described in Figure 3, and pore sealing condition is described in Figure 9.

To evaluate the formation of cracks after pore sealing semi-quantitatively, the total lengths of cracks, *L_c_*: m/m^2^, were measured on pure Al, 1050-Al alloy, 3003-Al alloy, and 5052-Al alloy (Figure 9 and Figure 10), and results are shown in Figure 15 (*L_c_* vs. *t_a_*). The crack length, *L_c_*, in anodic oxide films with *t_p_* = 1200 s, increases with *t_a_* on all the Al alloy specimens, while *L_c_* is zero throughout the anodizing time on pure Al. The tendency of the increase in *L_c_* with *t_a_* is more remarkable in the order of:

3003-Al alloy = 5052-Al alloy > 1050-Al alloy >> pure Al(5)

The width of cracks appears to be the largest on 3003-Al alloy, followed by 5052-Al alloy, and then 1050-Al alloy, in Figure 10. Replacing *L_c_* with the total surface area of cracks (*L_c_* × width), one can obtain the following order:

3003-Al alloy > 5052-Al alloy > 1050-Al alloy >> pure Al(6)

**Figure 16:**
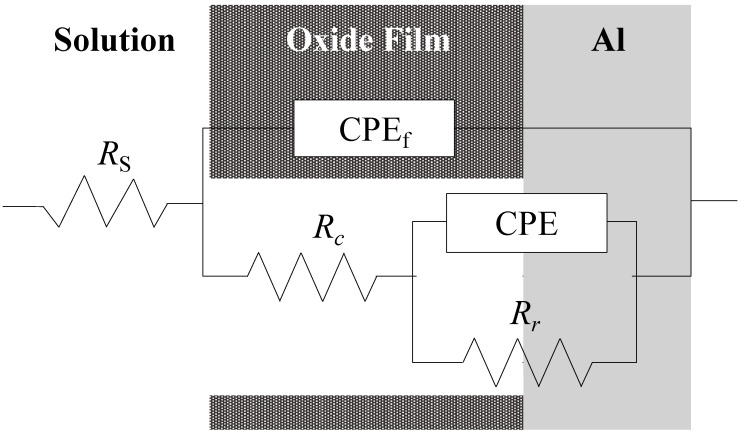
Equivalent circuit assumed from Figure 12. *R_s_*: resistance of bulk solution; *R_c_*: solution resistance in cracks; *R_c_*: reaction resistance at the bottom of cracks; *CPE_f_*: constant phase element of anodic oxide film; *CPE*: constant phase element at the bottom of cracks.

**Figure 17:**
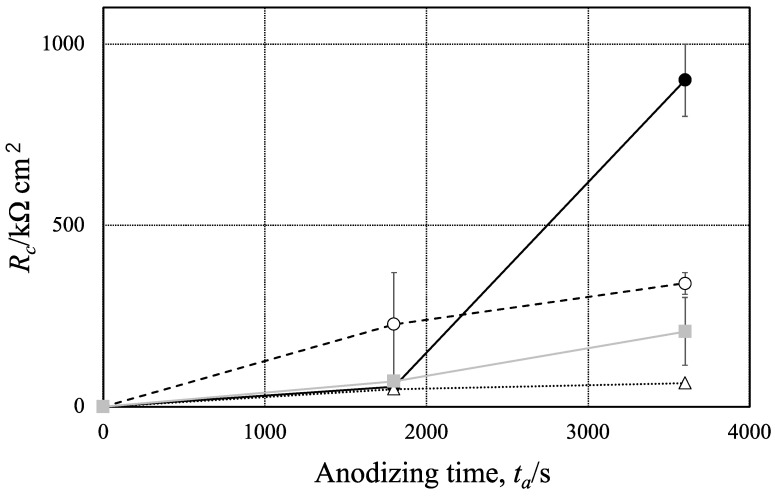
Relationship between solution resistance in cracks, *R_c_*, and anodizing time, *t_a_*, obtained for pure Al, 1050-Al alloy, 3003-Al alloy and 5052-Al alloy with *t_p_* = 1200 s. Anodizing condition is described in Figure 3, and pore sealing condition is described in Figure 9.

From the Bode and Nyquist Plots in Figure 11 and Figure 12, one can assume an equivalent circuit, as shown in Figure 16 [35,36,37,38]. In the equivalent circuit, *R_s_* is the resistance of bulk solution, *R_c_* the solution resistance in cracks, *R_r_* the rection resistance at the bottom of the cracks, *CPE_f_*, the constant phase element of anodic oxide film, and *CPE* the constant phase element at the bottom of the cracks. Figure 17 shows the relationship between *R_c_* and *t_a_*, obtained by the curve-fitting of Figure 12. The *R_c_* value increases with *t_a_* on all the specimens and the value at *t_a_* = 3600 s is in the order of:

Pure Al >> 1050-Al alloy > 5052-Al alloy > 3003-Al alloy(7)

The order in Equation (7) is opposite to that in Equation (6). This is reasonable because the *R_c_* value is inversely proportional to the exposed area of the substrate by the crack formation. 

Conclusively, pore sealing of anodized 1050-, 3003-, and 5052-Al alloys in boiling water only slightly improves the corrosion protection, while the corrosion protection is enormously improved by pore sealing on pure Al. This can be explained by the formation of cracks during pore sealing. Further investigation is necessary for the development of pore sealing without crack formation on Al alloys. 

## 5. Conclusions

In the present investigation, changes in the structure and corrosion protection ability of porous anodic oxide films on pure Al and Al alloys by pore sealing treatment, were examined, and the following were concluded.

(1) Pure Al after anodizing for 1800 and 3600 s has uniform porous oxide films with a smooth surface, but 1050-, 3003- and 5052-Al alloys have porous oxide films with imperfections, including pits and cavities.
(2) Pore sealing in boiling water leads to the formation of hydro-oxy-oxides in nano-pores of the porous oxide film uniformly on pure Al, and leads to the formation of cracks on the Al alloys.
(3) The total areas of cracks exposed increases with anodizing time on all Al alloys, and this is more remarkable in the order of 3003-Al alloy > 5052-Al alloy > 1050-Al alloy.
(4) The value of *R_c_,* evaluated by electrochemical impedance spectroscopy in 2 kmol m^−3^ -NaCl solution, increases with anodizing time on pure Al, and only slightly increases on Al alloys.

## Figures and Tables

**Table 1:** Chemical composition and thickness of pure Al and Al alloys specimens.

	Thickness of Specimen/mm	Components								
		Si	Fe	Cu	Mn	Mg	Zn	Cr	Ti	Al
Pure Al	0.50	0.01	0.00	0.01	0.00	0.00	0.00	0.00	0.00	Balance
1050-Al	0.30	0.10	0.36	0.02	0.00	0.00	0.01	0.00	0.00	Balance
3003-Al	0.28	0.27	0.57	0.14	1.19	0.00	0.03	0.00	0.00	Balance
5052-Al	0.97	0.10	0.10	0.02	0.04	2.59	0.01	0.20	0.01	Balance

**Figure A1:**
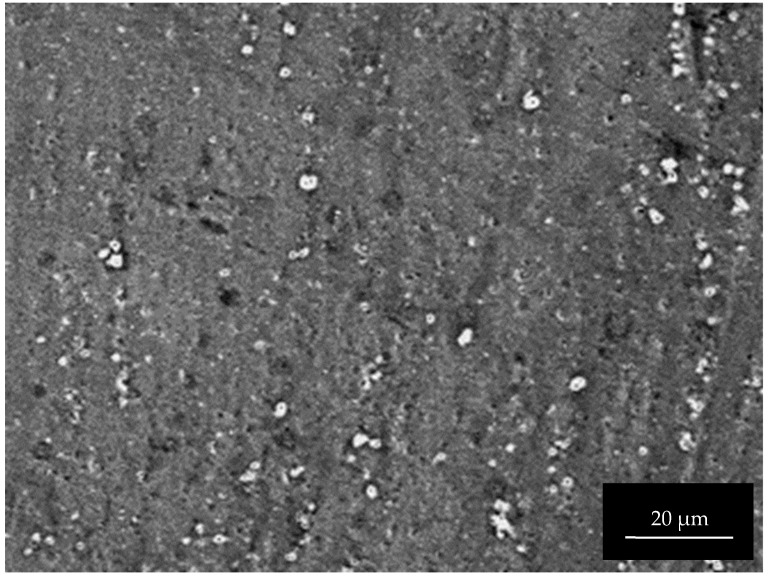
Surface of 3003-Al alloy with *t_a_* = 0. Figure 2c was enlarged to 500%.

**Figure A2:**
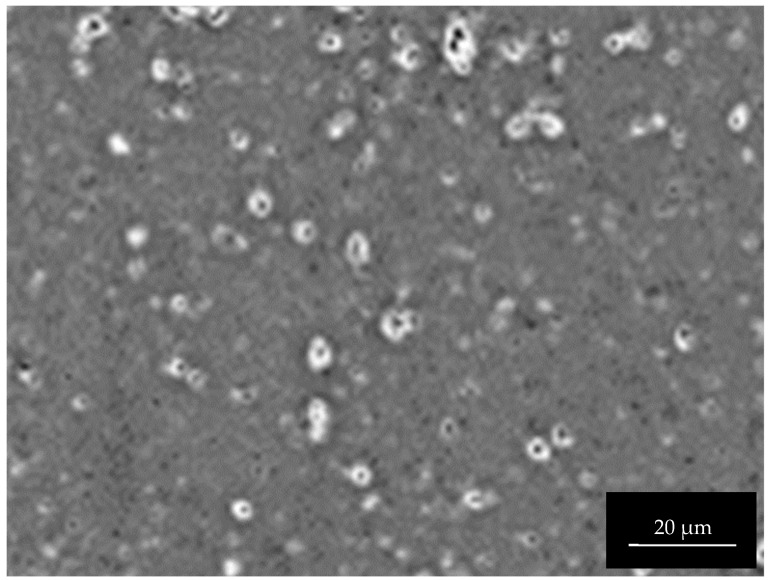
Surface of 3003-Al alloy with *t_a_* = 1800 s. Figure 4c was enlarged to 500%.

**Figure A3:**
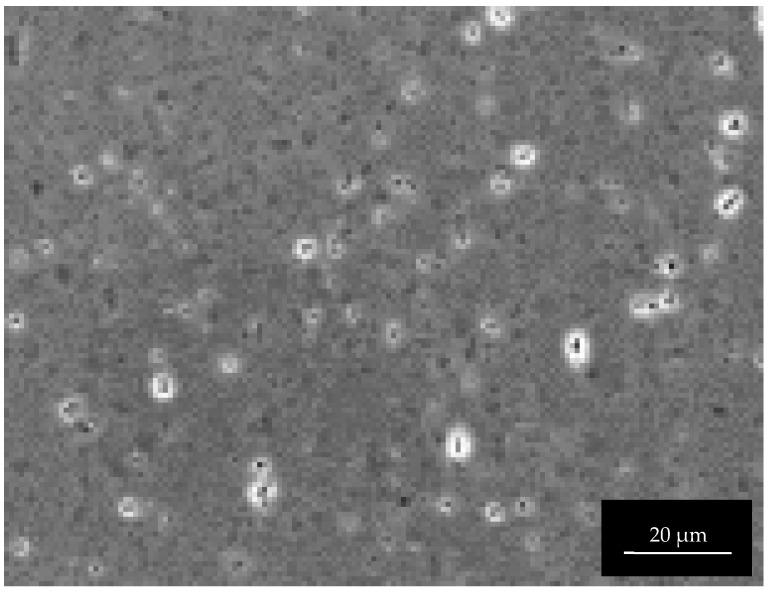
Surface of 3003-Al alloy with *t_a_* = 3600 s. Figure 5c was enlarged to 500%.

**Figure A4:**
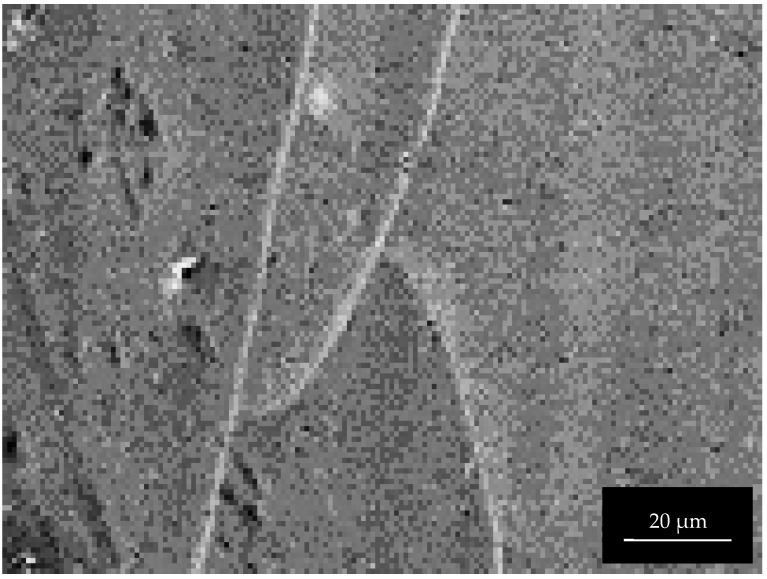
Surface of 3003-Al alloy with *t_a_* = 1800 s and *t_s_* = 1200 s. Figure 9c was enlarged to 500%.

**Figure A5:**
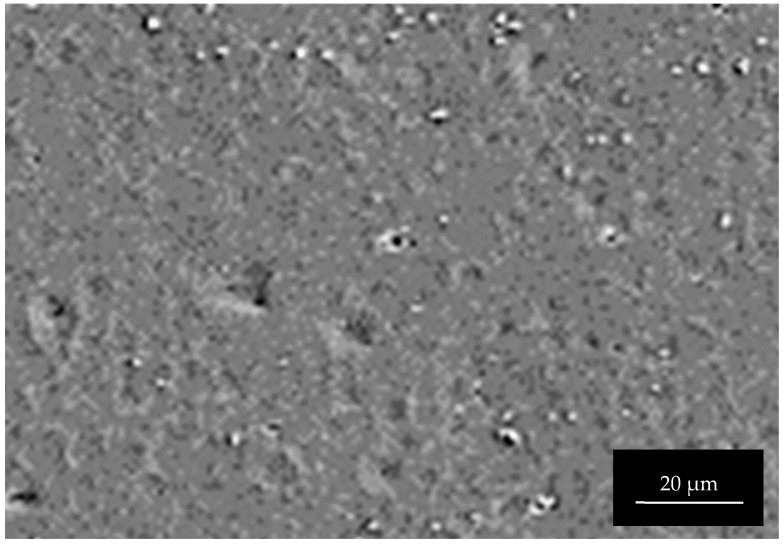
Surface of 1050-Al alloy with *t_a_* = 3600 s and *t_s_* = 1200 s. Figure 10a was enlarged to 500%.

**Figure A6:**
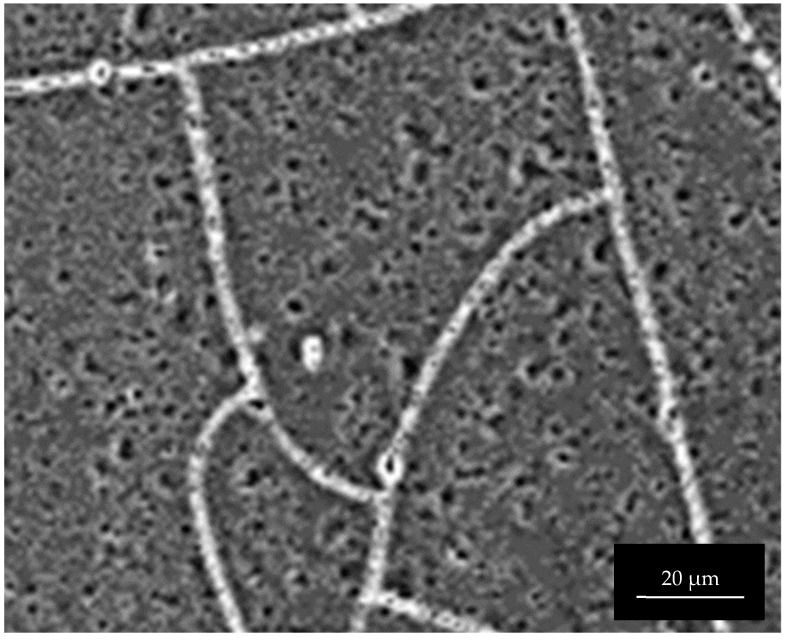
Surface of 3003-Al alloy with *t_a_* = 3600 s and *t_s_* = 1200 s. Figure 10c was enlarged to 500%.

## Data Availability

Not applicable.
